# Strategies and opportunities to STOP colon cancer in priority populations: pragmatic pilot study design and outcomes

**DOI:** 10.1186/1471-2407-14-55

**Published:** 2014-02-26

**Authors:** Gloria D Coronado, William M Vollmer, Amanda Petrik, Josue Aguirre, Tanya Kapka, Jennifer DeVoe, Jon Puro, Tran Miers, Jennifer Lembach, Ann Turner, Jennifer Sanchez, Sally Retecki, Christine Nelson, Beverly Green

**Affiliations:** 1 Kaiser Permanente Center for Health Research, Portland, USA; 2 Virginia Garcia Memorial Health Center, Portland, USA; 3 OCHIN, Portland, USA; 4 Group Health Research Institute, Seattle, USA

**Keywords:** Colorectal cancer screening, Fecal testing, Latinos, Hispanics, Safety net clinic, Federally qualified health center, Pragmatic study

## Abstract

**Background:**

Colorectal-cancer is a leading cause of cancer death in the United States, and Latinos have particularly low rates of screening. *Strategies and Opportunities to STOP Colon Cancer in Priority Populations* (STOP CRC) is a partnership among two research institutions and a network of safety net clinics to promote colorectal cancer screening among populations served by these clinics. This paper reports on results of a pilot study conducted in a safety net organization that serves primarily Latinos.

**Methods:**

The study assessed two clinic-based approaches to raise rates of colorectal-cancer screening among selected age-eligible patients not up-to-date with colorectal-cancer screening guidelines. One clinic each was assigned to: (1) an automated data-driven Electronic Health Record (EHR)-embedded program for mailing Fecal Immunochemical Test (FIT) kits (Auto Intervention); or (2) a higher-intensity program consisting of a mailed FIT kit plus linguistically and culturally tailored interventions delivered at the clinic level (Auto Plus Intervention). A third clinic within the safety-net organization was selected to serve as a passive control (Usual Care). Two simple measurements of feasibility were: 1) ability to use real-time EHR data to identify patients eligible for each intervention step, and 2) ability to offer affordable testing and follow-up care for uninsured patients.

**Results:**

The study was successful at both measurements of feasibility. A total of 112 patients in the Auto clinic and 101 in the Auto Plus clinic met study inclusion criteria and were mailed an introductory letter. Reach was high for the mailed component (92.5% of kits were successfully mailed), and moderate for the telephone component (53% of calls were successful completed). After exclusions for invalid address and other factors, 206 (109 in the Auto clinic and 97 in the Auto Plus clinic) were mailed a FIT kit. At 6 months, fecal test completion rates were higher in the Auto (39.3%) and Auto Plus (36.6%) clinics compared to the usual-care clinic (1.1%).

**Conclusions:**

Findings showed that the trial interventions delivered in a safety-net setting were both feasible and raised rates of colorectal-cancer screening, compared to usual care. Findings from this pilot will inform a larger pragmatic study involving multiple clinics.

**Trial registration:**

ClinicalTrial.gov: NCT01742065

## Background

Colorectal-cancer is the second leading cause of cancer death in the US; the Surveillance, Epidemiology and End Results program (SEER) predicts that, in 2013, there will be 142,000 new cases and 51,000 deaths from colorectal-cancer [1]. While regular colorectal-cancer screening has been shown to reduce colorectal-cancer mortality [2], screening rates are low in the general population, and particularly low in certain population subgroups. Data from the Behavioral Risk Factor Surveillance System from 2012 show that 53% of Latinos ages 50–74 were current with colorectal-cancer screening recommendations compared to 66% of non-Latino whites [3]. Colorectal-cancer screening rates are also low among those who lack health insurance (37% vs. 69% among those with insurance) or who lack a regular source of health care (31% vs. 69% among those with a regular source of care) [3].

Previous evaluations of clinic-based programs to improve rates of colorectal-cancer screening have shown that direct mailing of fecal occult blood tests (gFOBT) or fecal immunochemical tests (FIT) consistently led to 6–24% increases in colorectal-cancer screening regardless of clinical setting [4-7]. Interventions that included patient navigators (staff trained to promote screening completion and provide on-going communications and assistance with overcoming barriers) were also consistently effective and mainly focused on underserved populations [5,6,8-11]. Use of health educators and screening information tailored to specific cultural and language needs have been effective in some studies [4,6,9-11]. Although some showed promising results, none of the previous interventions embedded their registry functions directly into the electronic health record (EHR), and into existing clinical staff workflows.

Our team had previously tested two direct-mail colorectal-cancer screening programs in clinical settings. One pilot tested the program among 500 low-income Latinos, but relied on manual medical chart review to identify patients and track screening outcomes [4]. A second tested a randomized controlled trial in a Health Maintenance Organization (HMO) that used an EHR-linked system for patient identification and tracking, but was managed by a research team [5]. Both resulted in a 24% increase in colorectal-cancer screening, over usual care.

As part of a large multi-site pragmatic study to test automated strategies to raise the rates of colorectal-cancer screening in safety-net clinics, we pilot-tested two clinic-based interventions in a single safety-net clinic organization (comprised of 4 clinics). The Auto Intervention consisted of an automated data-driven, EHR-embedded program for mailing FIT kits to patients due for colorectal-cancer screening. The Auto Plus Intervention is a higher-intensity program consisting of the same intervention as the Auto clinic, plus linguistically and culturally tailored interventions that account for the clinics’ resources, capacity, and preferences. For the pilot, the additional intervention chosen by the clinic was live telephone counselling that used motivational interviewing techniques. The pilot study involved a partnership with Virginia Garcia Memorial Health Center (VGMHC), a federally qualified health center (FQHC) that operates a network of 4 primary care clinics in the Portland, Oregon, metropolitan area and specializes in the culturally competent delivery of primary care services to low-income patients, particularly Latinos. The pilot sought to implement the program using existing EHR tools and clinic personnel; to assess the feasibility of disseminating it to a large network of clinics; and to report preliminary estimates of the interventions’ effectiveness and reach, based on aspects of the RE-AIM framework [12]. For this report, we focus on quantitative data only; findings from qualitative interviews with patients and clinic staff will be reported separately.

## Methods

Strategies and Opportunity to STOP Colon Cancer in Priority Populations (STOP CRC) is a Demonstration Project of the National Institutes of Health (NIH) Health Care Systems Research Collaboratory [UH2AT007782]. The Collaboratory seeks to strengthen national capacity to implement cost-effective large-scale pragmatic studies that engage health care delivery organizations as research partners, recognizing that such partnerships are essential to strengthen the relevance of research results to health practice. As such, STOP CRC is a pragmatic study [13]; this meant that we designed our program so that it could be incorporated into clinical practice; we allowed the clinic to choose intervention components, and we worked with existing clinic staff and infrastructure. All study procedures were reviewed and approved by the Institutional Review Board of Kaiser Permanente Northwest (#3397), which is in compliance with the Helsinki Declaration.

### Setting and background

In Oregon, the Latino population represents 12% of the total state population. Latinos are the fastest growing population in the state, having increased by 64% (174,748 individuals) between the 2000 and 2010 censuses [14]. Many Latino patients in Oregon receive care at either FQHCs or “look-alikes” (serving similar populations), referred to collectively as safety-net clinics. Our partnering FQHC, VGMHC, specializes in services to Latino patients. In 2012, VGMHC had 5,190 active patients aged 50–74, of whom 46% were Latino and 59% were uninsured. Data from 2012 show that the overall rate of fecal testing (gFOBT or FIT) at VGMHC was 5.1%. For this project, VGMHC chose to use OC Micro (PolyMedco, Inc, New York), a one-sample FIT kit, and to process it at a commercial laboratory. To assure follow-up colonoscopy services for low-income patients, VGMHC partnered with Project Access Now, a local community organization that connects low-income, uninsured individuals to donated specialty medical services, including diagnostic colonoscopy, through a coordinated network of volunteer providers.

To aid with the process of incorporating the intervention into clinical practice and cultural relevance, we convened a community advisory board; the board consisted of policy-makers, clinicians, patients and their advocates, and gastroenterologists. The board met 5 times throughout the year during a single 4-hour in-person meeting and 4–1.5 hour phone meetings. We also held regular meetings of project investigators and clinic staff.

### Participants

The pilot study aimed to recruit 200 patients aged 50–74, who received care in the past year at either of the two participating intervention clinics of VGMHC, and who were not up-to-date with recommendations for colorectal-cancer screening (did not have a gFOBT/FIT in the past 11 months, a colonoscopy in the past 9 years, or a sigmoidoscopy in the past 4 years). Consistent with the pragmatic nature of the STOP CRC study, otherwise eligible patients were excluded only if they had a history of colorectal disease, a significant co-morbid condition, or a referral to gastroenterology in the past year. To minimize staff training at each site, patients were selected from a single provider team at each of the intervention sites. We chose to include patients whose primary language was English or Spanish, to allow assessment of our cultural adaptations.

### Stratification

To assess the feasibility and effectiveness of our program in various subgroups, eligible patients were randomly selected across three stratification variables. These variables were insurance status (insured vs. uninsured), preferred language (Spanish vs. English) and date of most recent clinic visit (< 3 months vs. > = 3 months). Within each clinic, eligible patients were grouped by stratification variable (total n groups = 8), and up to 16 patients were randomly selected within each group. Group sizes ranged from 6 to 16 patients.

### Interventions

The intervention compared patients enrolled in clinics using two different approaches to raising rates of colorectal-cancer screening—Auto Intervention or Auto Plus Intervention—with patients enrolled in a clinic assigned to usual care. Our goal was to inform the design of a future larger pragmatic study involving multiple safety-net clinics. Clinic staff were trained to use the system by the EHR Site Specialist who had helped design the system; the Patient Care Coordinator at the Auto Plus clinic received bilingual motivational interviewing training from a bilingual project staff.

### Usual care

For the purposes of this pilot, a single clinic in the VGMHC network was identified to serve as the usual-care site. Usual care entailed the receipt of any information and outreach on the importance of colorectal-cancer screening and ordering of screening tests provided routinely by clinic staff on an opportunistic basis during routine clinic encounters for age-eligible patients. The two interventions, implemented at separate clinic sites, were overlaid on usual care offered at each clinic.

### Mailed FIT kit (Auto Intervention)

The Auto Intervention consisted of an automated data-driven, EHR-embedded program for mailing FIT kits (with linguistically appropriate pictographic instructions and return postage) to patients due for colorectal-cancer screening. Eligible patients, based on inclusion/exclusion criteria described above, were sent an introductory letter (written in English and Spanish) explaining the STOP CRC study and offering patients an opportunity to opt out. Patients whose introductory letters were not returned by the Post Office were presumed to have a valid address, and were mailed a FIT kit and bilingual instructions for completing the FIT. Patients who failed to return a completed FIT kit within three weeks were mailed a bilingual reminder postcard.

### Mailed FIT kit plus outreach (Auto Plus Intervention)

The Auto Plus Intervention was a higher-intensity program consisting of the same intervention as the Auto clinic, plus linguistically and culturally tailored interventions delivered at the clinic level that account for individual clinics’ resources, capacity, and preferences. For the pilot, the additional intervention chosen by the clinic was live telephone counselling that made use of motivational interviewing techniques, delivered in English or Spanish by the team’s bilingual Patient Care Coordinator. Patients who were identified as eligible for colorectal-cancer screening were mailed an introductory letter, FIT kit, and reminder postcard as described in the Auto Intervention. Patients who failed to return the FIT kit after 1 month of the mailed reminder postcard were eligible for live telephone counselling, and all received at least 2 phone attempts.

### Pilot outcomes

The primary purpose of the pilot was to assess the feasibility of conducting an EHR-enabled colorectal-cancer screening intervention that could be scaled up to multiple safety net clinics. We were interested in two aspects of feasibility: (1) whether our colorectal-cancer screening registry function could be embedded directly into the EHR and use real-time data to identify patients eligible for each intervention step; and (2) whether affordable testing and follow-up care to uninsured patients could be provided, given our qualitative findings documenting low provider recommendations for colorectal-cancer screening due to such barriers. Components of the RE-AIM framework guided other aspects of our feasibility assessment [12]. Specifically, as outlined in the framework, we were interested in whether clinic staff would successfully deliver each component of the intervention (implementation), in the proportion of patients we could successfully contact (reach), and in the proportion that would complete testing (effectiveness). We were also interested in whether certain population subgroups would be more or less responsive to our intervention. Given the nature of our design, we were unable to assess two aspects of the RE-AIM framework: adoption and maintenance.

### Statistical analysis

Preliminary estimates of effectiveness were obtained and serve as point estimates for sample sizes needed for our planned multi-site pragmatic study using a cluster randomized design. EHR data was used to calculate the proportion of FIT kits returned within 6 months of the initial mailing for the Auto and Auto Plus intervention clinics; these proportions were compared with similar data from the usual care clinic. The date of hypothetical “rollout” (*i.e.,* initial mailing) for the usual-care site was timed to coincide with the rollout dates for the intervention sites. The measurement period was from 1/18/2013 to 7/17/2013.

Reach was assessed by calculating the delivery of each program component (*i.e.,* N intro letters mailed/N anticipated, N kits mailed/N anticipated; N reminder postcards mailed/N anticipated; N phone call delivered/N anticipated). Consistent with the pilot nature of this study, all analyses were descriptive in nature. Our focus was on describing intervention process data and estimating gFOBT/FIT completion probabilities for the two intervention clinics overall and among selected subgroups.

## Results

### Feasibility

We were able to build an EHR-embedded program that used real-time data to identify eligible patients at each step in our intervention and to track colorectal-related outcomes. Our intervention was delivered to all anticipated patients at each step (implementation). Our assessment of reach showed that the STOP CRC intervention could be delivered to a high proportion of intended patients (*i.e.,* in only 7.5% of households were letters or kits retuned by the Post Office, and a live phone call to conduct a motivational interview was completed for 53% of the patients in the Auto Plus Intervention group who were eligible for that step). Notably, consistent with the pragmatic nature of our design, clinic staff followed the usual clinic procedure of making 2 attempts to reach a patient by phone. The clinic chose to pay for testing in uninsured patients, which meant that additional arrangements were made with the outside lab, so that patients with insurance could be billed directly and those without could be billed to the clinic. A local community organization that provides specialty services to uninsured patients in the Portland Metro area, Project Access Now, agreed to provide colonoscopies to uninsured patients with abnormal test results. Staff at participating clinics adapted existing workflows for use in the STOP CRC project. The staff were successfully trained in the use of the EHR tools. Notably, the pilot involved a one-time selection of eligible patients and mailing of outreach materials.

### Participant selection

**Figure 1:**
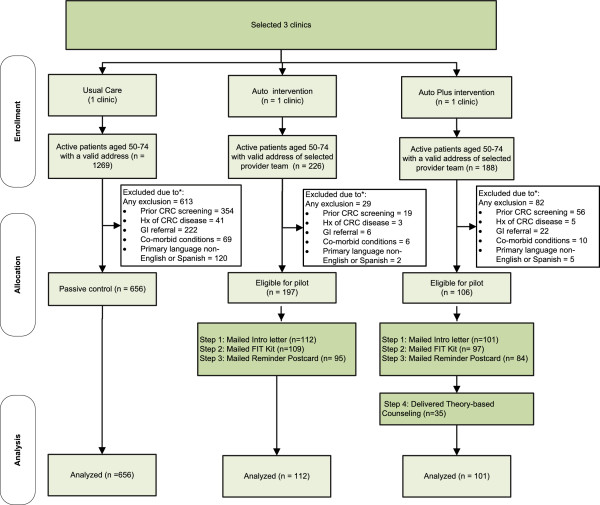
CONSORT diagram of STOP CRC pilot.

A total of 226 patients in the Auto Clinic, and 188 patients in the Auto Plus Clinic, were initially identified as active patients aged 50–74 who had a valid address (Figure 1). After exclusions, 197 and 106 were eligible for the pilot; we randomly selected 213 patients (112 patients in the Auto Clinic and 101 in the Auto Plus Clinic) based on our stratification variables.

For the passive control clinic, a total of 1,269 were initially identified as active patients aged 50–74 who had a valid address. After exclusions, 656 patients were eligible and included in our analysis.

Selected participants were generally aged 50–64 (82%), female (62%), Hispanic (49%), and uninsured (44%), and had household incomes below 100% of the Federal Poverty Level (81%); 44% reported Spanish as their preferred language (Table 1).

**Table 1:** Characteristics of sample

	**Auto clinic**		**Auto plus clinic**		**Usual care clinic**	
**Characteristic**	**(n = 112)**		**(n = 101)**		**(n = 656)**	
	**N**	**%**	**N**	**%**	**N**	**%**
**Age**						
50–64	85	75.9	84	83.2	545	83.1
65–74	27	24.1	17	16.8	111	16.9
**Gender**						
Female	75	67.0	60	59.4	405	61.7
Male	37	33.0	41	40.6	251	38.3
**Ethnicity**						
Hispanic	71	63.4	54	53.5	297	45.3
Non-Hispanic	41	36.6	32	31.7	282	43.0
Unknown	0	0.0	15	14.9	77	11.7
**Language**						
English	45	40.2	45	44.6	391	59.6
Spanish	67	59.8	56	55.4	265	40.4
**Insurance status**						
Medicaid/Medicare	50	44.6	42	41.6	304	46.3
Uninsured	49	43.8	55	54.5	280	42.7
Commercial	13	11.6	4	4.0	72	11.0
**Federal poverty level**						
<100%	85	75.9	90	89.1	531	80.9
100–150%	21	18.8	8	7.9	73	11.1
151%+	6	5.4	3	3.0	52	7.9
**Number of visits in past year**						
1	24	21.4	19	18.8	165	25.1
2–5	64	57.1	58	57.4	337	51.4
6 +	24	21.4	24	23.8	154	23.5

### Receipt of STOP CRC program (program reach)

A total of 213 participants (112 in the Auto Clinic and 101 in the Auto Plus clinic) were mailed an introductory letter (Table 2). The FIT kit was mailed to 206 patients (109 in the Auto and 97 in the Auto Plus). A total of 179 patients were mailed a reminder postcard. For the follow-up phone calls in the Auto Plus clinic (anticipated n = 66), 30 (53%) were reached and counseled; the remaining 31 were not reached (20), declined (4) or had a disconnected/wrong number or moved (7). An introductory letter or FIT kit was returned as undeliverable for 16 participants (7.5%), addresses for 11 of these were updated and FIT kits were re-sent.

**Table 2:** Intervention activities delivered

		**Auto clinic**	**Auto-plus clinic**	**Usual care clinic**
**Step 1:**	**Introductory letters mailed**	112	101	656
	Invalid address*	3	1	--
	Opted out	0	3	--
**Number eligible for mailed kit**		109	97	
**Step 2:**	**Kits mailed**	109	97	--
	Invalid address*	1	0	--
	Opted out	1	0	--
	Completed FITs	12	13	--
**Number eligible for reminder postcard**		95	84	
**Step 3:**	**Reminder postcards mailed**	95	84	**--**
	Completed FITs	32	18	--
**Number eligible for theory-based phone counseling**			66	
**Step 4:**	Completed call**	NA	30	--
	Wrong/disconnected number/moved	NA	7	--
	Not reached	NA	20	--
	Opted out	NA	4	--
	Complete FITs	NA	5	--
**Total screened**				
Total FIT/FOBTs		44 (39.3%)	37 (36.6%)***	7 (1.1%)
Total Colonoscopy		5 (4.5%)	3 (3.0%)	5 (0.7%)

*16 letters or FIT kits were returned, when possible, addresses were updated or patients were called and re-sent a FIT kit.

**Completed call includes patients who requested a new kit, or indicated that their test was in process.

***Includes 1 FOBT completed as part of usual care (not mailed by intervention).

### Receipt of CRC screening

Of the 213 patients who were originally selected, 44 and 37 patients in the Auto and Auto Plus clinics, respectively, mailed back their FIT for processing (for an intention–to-treat effect size of 39.3% in Auto and 36.6% in Auto Plus). The rate of fecal testing in the 656 patients in the usual care clinic over the same time period was 1.1% (Table 2). A total of 13 patients were referred for colonoscopy during this time period; 4.5% in the Auto clinic; 3.0% in the Auto Plus clinic, and 0.7% in the usual care clinic. Intervention clinic screening rates appeared to differ by demographic characteristics, with the highest rates observed among the 65 – 74 age group, Hispanics, and those whose primary language was Spanish (Table 3). Among the 81 patients tested, 7 were found to have a positive test result and all were referred for follow-up colonoscopy, and all but one completed colonoscopy (1 patient declined). No serious adverse events were reported related to the study.

**Table 3:** Fecal test completion by demographic characteristic and health care utilization

	**Auto clinic**		**Auto plus clinic**		**Usual care clinic**	
**Characteristic**	**Total (n = 112)**	**Completer (n = 44)**	**Total (n = 101)**	**Completer (n = 37)**	Total* (n = 656)	**Completer (n = 7)**
	**N**	**N (%)**	**N**	**N (%)**	**N**	**N (%)**
**Age**						
50–64	85	31 (36.5)	84	29 (34.5)	545	5 (0.9)
65–74	27	13 (48.1)	17	8 (47.1)	111	2 (1.8)
**Gender**						
Female	75	30 (40.0)	60	25 (41.7)	405	5 (1.2)
Male	37	14 (37.8)	41	12 (29.3)	251	2 (0.8)
**Ethnicity**						
Hispanic	71	31 (43.7)	54	27 (50.0)	297	4 (1.3)
Non-Hispanic	41	13 (31.7)	32	7 (21.9)	282	2 (0.7)
Unknown	0	0 (0.0)	15	3 (20.0)	77	1 (1.3)
**Language**						
English	45	15 (33.3)	45	10 (22.2)	391	3 (0.8)
Spanish	67	29 (43.3)	56	27 (48.2)	265	4 (1.5)
**Insurance status**						
Medicaid/Medicare	50	20 (40.0)	42	13 (31.0)	304	2 (0.7)
Uninsured	49	17 (34.7)	55	23 (41.8)	280	5 (1.8)
Commercial	13	7 (53.8)	4	1 (25.0)	72	0 (0.0)
**Federal poverty level**						
<100%	85	31 (36.5)	90	33 (36.7)	531	7 (1.3)
100–150%	21	10 (47.6)	8	4 (50.0)	73	0 (0.0)
151+%	6	3 (50.0)	3	0 (0.0)	52	0 (0.0)
**Number of visits in past year**						
1	24	6 (25.0)	19	2 (10.5)	165	0 (0.0)
2–5	64	23 (35.9)	58	29 (50.0)	337	6 (1.8)
6 or more	24	15 (62.5)	24	6 (25.0)	154	1 (0.6)

## Discussion

The STOP CRC study Auto and Auto Plus interventions were successfully implemented in two safety-net clinics. Both interventions led to higher colorectal-cancer testing rates than rates in the usual care clinic, demonstrating the effectiveness of an EHR-embedded intervention addressing colorectal-cancer screening. Our pilot findings showed high reach for the mailed component (based on the low number of mailed items that was returned from the Post Office), and moderate reach for the phone-call component (based on 2 call attempts). Further research is needed to assess effectiveness of the program as an on-going part of standard clinical care (not as a one-time mailing), and to assess the adoption, implementation, and maintenance of the program. If successful, the program may represent an effective method of raising levels of participation in colorectal-cancer screening and improving earlier-stage detection of colorectal-cancer among patients least likely to be screened.

Our findings showed substantially higher colorectal-cancer testing rates in our two interventions clinics, compared to similar patients in a third VGMHC clinic that did not receive the intervention. The differences in rate of fecal testing in our two intervention sites versus the usual care site (difference in Auto Clinic vs. Usual care: 38% and difference in Auto Plus Clinic vs. Usual care: 35%) were higher than effect sizes observed in previous clinical studies on the same topic [4-7].

Our point estimate for differences in fecal testing rates between our Auto and Auto Plus clinics was marginal (Difference in differences: 38% - 35% = 3%). This may be due, in part, to the lower response in the Auto Plus clinic to the mailing of the introductory letter, and reminder postcard (FIT return rate: 32%), compared to the Auto Clinic (39%). Of the 66 Auto Plus patients identified for theory-based phone counseling, 8% of those identified, and 17% of those successfully reached, returned their FIT kits. Pooling our FIT completion rates for the 2 clinics, our best estimate of effectiveness of the Auto intervention alone is 36%, plus another 2% from phone-based follow-up. This is consistent with findings from 3 studies that used telephone reminders or theory-based phone counselling [4,15,16], but differed from a study conducted by Green et al. at Group Health Cooperative, which showed an added bump of 7 percentage points associated with brief phone assistance, and a further bump of 7 percentage points with more intensive ongoing phone-based navigation [5]. It is important to note that Green et al. used medical assistants and/or nurses who were hired by the study to deliver the interventions, whereas STOP CRC integrated intervention delivery into routine care. We cannot rule out the possibility that the apparent lack of effect of the phone counseling in our pilot was due to small sample sizes or differences in baseline characteristics of clinics or selected patients.

Our observation that only 16/213 (7.5%) participants were found to have an invalid address (as determined by their introductory letter or kit being returned by the Post Office) was contrary to expectation. This may be due, in part, to a system-wide mailing to update patient address information that took place 3 months before our introductory letter was sent. Notably, while we observed high reach for our mailed components, it is plausible that some mailings were not received by their intended participants. Also, we anticipate that clinics with less up-to-date patient address information will achieve lower reach.

While our sample size is too small to permit statistical comparisons across subgroups, our pilot data are suggestive of high levels of effectiveness among Hispanics and other individuals who speak Spanish. Notably, among Auto Clinic patients, the highest rate of fecal testing was found among those who had 6 or more clinic visits; this suggests that personal interactions with a provider in addition to the mailed program may serve to reinforce the importance of screening. This finding is consistent with data from Liles et al. in a study that enrolled patients at Kaiser Permanente Northwest [17].

Our pilot program has some limitations that we plan to address in the larger multi-site study. Our inclusion and exclusion criteria rely on EHR data, and we could not verify the accuracy of colonoscopy receipt, raising the possibility that our intervention was delivered to patients who were ineligible due to recent colorectal-cancer screening. Nevertheless, a minority of patients opted out (n = 8), and only 3 opted out because of prior testing. We plan to address this by conducting a robust validation of EHR codes used for our inclusion and exclusion of participants for the larger study. We also plan to enhance the capture of colorectal-cancer screening in EHR-based tools for tracking outside screening events (called Health Maintenance in Epic). Our feasibility assessment relied on quantitative data only; we plan to report separately on feasibility considerations based on qualitative interviews with providers and patients. Moreover, we report no data on the cost of providing affordable testing and follow-up care for patients in this setting, which may drive feasibility and sustainability over time.

The small size and non-random nature of our sample limit the interpretation of our findings. Intervention effects are inextricably confounded with clinic effects, and the interventions were delivered only to patients in the practices of a single team (2–3 providers and their support staff of a registered nurse, patient care coordinator, and team assistant processing referrals) in each clinic. The patient panels appeared to differ with regard to the proportions that were excluded because of prior colorectal-cancer screening and other factors. These providers volunteered for the intervention and may have been more willing to involve their staff in conducting follow-up calls than providers in the clinic as a whole. Nevertheless, because the 3-sample gFOBT cards, and not the FIT, were offered during clinic encounters as part of usual care, we could easily discern that our findings were not impacted by more frequent recommendations for screening during clinic encounters. Nevertheless, the differences in screening probabilities between intervention and usual-care clinics were striking and we will use them to help inform power calculations for the larger study.

Our pilot provided some important information that will inform the design of a large-scale pragmatic study to test the effectiveness of the program in multiple safety-net clinics. We report successful implementation, high reach for mailed components, moderate reach for telephone components, and high effectiveness for both interventions. We were also able to successfully embed our registry tools into the EHR, and use real-time data to identify patients eligible for each intervention step.

These findings, as well as findings from on-going analysis of qualitative interviews with patients and providers, will inform several aspects of a planned multi-clinic study that will enroll a broad range of patients. Specifically, our preliminary estimates of effectiveness suggest that additional telephone-based outreach may not be needed. Further exploration of how a variety of factors may influence preventive services use may be needed to inform further refinements to the program.

## Conclusion

Our STOP CRC pilot shows the great potential of a larger-scale intervention to reduce disparities in colorectal-cancer screening and push back stage of detection through improved uptake of colorectal-cancer screening in a population that has historically had low colorectal-cancer screening rates. Our pilot study also demonstrated the feasibility of conducting an EHR-based direct-mailed colorectal-screening intervention at two clinics and of working with clinic staff to deliver the intervention elements.

## Competing interests

The authors declare that they have no competing interests.

## Authors’ contributions

GC drafted and revised the manuscript; GC, BG, and JD led the study; WV designed the analytic plan and oversaw the statistical analyses; AP conducted the statistical analysis; all authors contributed to the iterative process of engaging clinic stakeholders to develop effective EHR-based tools to facilitate the intervention, and JA provided training to clinic staff in how to use the tools; and JS provided bilingual training in motivational interviewing. CN and SR provided guidance on clinic interactions and SR led the Advisory Board for this project. All authors read and approved the final manuscript.

## Pre-publication history

The pre-publication history for this paper can be accessed here:

http://www.biomedcentral.com/1471-2407/14/55/prepub
